# Cycle of violence in women victims of domestic violence: Qualitative analysis of OPD 2 interview

**DOI:** 10.1002/brb3.1430

**Published:** 2019-10-06

**Authors:** Luciane Maria Both, Taís Cristina Favaretto, Lúcia Helena Machado Freitas

**Affiliations:** ^1^ Federal University of Rio Grande do Sul (UFRGS) – CAPES Scholarship Porto Alegre Brazil; ^2^ Federal University of Rio Grande do Sul (UFRGS) Porto Alegre Brazil; ^3^ Department of Psychiatry and Legal Medicine Psychiatry Service of Federal University of Rio Grande do Sul (UFRGS)/Hospital de Clínicas de Porto Alegre (HCPA) Porto Alegre Brazil; ^4^ Center of Treatment and Studies of Psychic Trauma Psychiatric Service of Hospital de Clínicas de Porto Alegre (HCPA) Porto Alegre Brazil

**Keywords:** cycle violence against women, domestic violence, psychodynamics, psychological trauma, qualitative research

## Abstract

**Introduction:**

Domestic violence places woman as the victim and man as the aggressor in the family environment. There is limited consistent and clear information based on empirical evidence on the dynamic functioning of the victims.

**Objective:**

To further understand the psychodynamics of women in the cycle of violence taking into account the aspects of psychological trauma. It is transversal research design. The sample was composed of ten women victim of domestic violence. Data collection was based on the OPD‐2 Clinical Interview. Content analysis was performed from categories created by a posteriori: (a) Previous history; (b) Behavioral aspects; (c) Emotional aspects; (d) Reason for being in the relationship; (e) Type of violence and explanation for the reason of violence; (f) Support network and daily activities; and (g) Clinical and legal referral.

**Results:**

Constant violence causes changes in the structural functioning and psychological conflict of the victims: difficulties in mentalization, instability in relationships, emotional dependence, abandonment of her own life for her partners, difficulty in having a sense of identity. Victims presented difficulties in making significant changes in daily life to break the cycle of violence.

**Conclusion:**

The research sought to collaborate with more evidence on the subject, suggesting a reformulation on forms of encounter to break the cycle of violence.

## Graphical abstract

Figure 2 shows the cycle of violence.

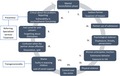

## 1 INTRODUCTION

Violence refers to the concept of power and the use of superiority over the other (Minayo et al., 2018). More specifically, domestic violence places women as victim and man as the aggressor and there is damage or lack of physical and psychological well‐being—Maria da Penha Law (Brazil, 2006; Cortez, Souza, & Queiróz, 2010): physical violence, psychological violence, or sexual violence. It is considered a subcategory of gender violence (Lourenço et al., 2013) and a serious social phenomenon to be combated by public health (Bins, Telles, & Panichi, 2015; Rafael & Moura, 2013) and by human rights services around the world. However, it is a challenge to the healthcare sector due to the high statistical incidence and severe outcomes (Osis, Duarte, & Faúndes, 2012). In Brazilian context, there is trivialization and even acceptance of violent behavior against women in some subcultures of society (Bins et al., 2015; Falcke & Féres‐Carneiro, 2011; Minayo et al., 2018).

Statistical data show that 23% of women suffer from their partners (WHO, 2019). The global prevalence of domestic violence estimates that approximately 30% of women suffer this type of violence throughout their lives (WHO, 2013). Also, violence against women is calculated by the number of female homicides committed by their partners—uxoricides. In 2017, it was estimated that 2,795 women were victims of femicide in the 23 countries of Latin America and the Caribbean. These are data from the latest report of the recently published Gender Equality Observatory of the Economic Commission for Latin America and the Caribbean (ECLAC). Brazil holds the unfortunate record of the highest absolute number of femicides, with 1,133 victims confirmed in 2017 (WHO, 2019). According to data from the Public Security Secretariat of Rio Grande do Sul (2014), at the end of 2014 there were 75 women murdered by their partners and 287 attempts. No data were reported on the homicide of men committed by their partners. The capital city, Porto Alegre was number 1 in the ranking of women victim in the state.

As a whole, violence can cause serious trauma to the victim. Psychological trauma is considered to be the results of physical and/or psychological threat (Eizirik et al., 2006; Peres, 2009). The way each subject deals with and represents the traumatic events is linked to the intensity and severity of the incident. Thus, the violent act felt as trauma triggers preexisting conflicts (Garland, 2015). In this way, care for the trauma is mainly provided by the family, primary caregivers, assisting in the structuring of safer and more stable subjects (Winnicott, 1993). With a safe foundation, the subject will develop a capacity for mentalization—the ability to discriminate internal and external aspects of reality and to understand self and others' mental states and the capacity for emotional regulation, considered vital for the organization of self (Bateman & Fonagy, 2016; Fonagy & Allison, 2012).

The psychoanalytic‐oriented treatment for victims of domestic violence is considered a challenge due to the adherence of the victim and the countertransference of the professional. Dealing with strong emotional demands from the violent events, as well providing competent treatment, can be difficult for the therapist. Thus, a consistent and clear understanding based on empirical evidence on the dynamic functioning to focus on pertinent and specific issues of this population becomes extremely important. These victims are characterized by constant traumatic situations that affect psychological and emotional functioning (Crempien, 2009). In this sense, there is a lack of studies with psychodynamic focus in the domestic violence context.

Dynamics of domestic violence implies repetitive behavioral patterns in relationships, maintaining the cycle of violence. Usually, it is presented with a slow and silent beginning without physical aggression, gradually progressing to actions with greater intensity to humiliation beatings, as well as even public manifestations of aggression. Moreover, it can be aggravated by women's shame when reporting violence, lack of educational means and access to legal information and poor assistance and protection (Falcke, Boeckel, & Wagner, 2017). Moreover, why do these women remain in this cycle of violence? What does it take to break it? From the diversity present in gender studies, we consider it fundamental to highlight their involvement in maintaining the cycle of violence.

Therefore, the present study has the objective to increase the psychodynamical understanding of woman living in cycle of violence, considering the psychological trauma as an important part of the abusive situation, more specifically, to explore the previous history, the behavioral and emotional aspects involved, the reasons why they stay and allow the perpetuation of the cycle of violence, the existence of social network, the meanings attributed to violence, and possible referrals.

## 2 METHOD

This is a qualitative and transversal study whose focus was the content analysis of the interviews. The construction of the study was based on the Consolidated Criteria for Reporting Qualitative Research (COREQ; Tong, Sainsbuty, & Craig, 2007). Finally, this research is part of a larger study project of violence against Brazilian women.

### 2.1 Participants

Ten women victims of domestic violence participated in this study. They had medical examination for legal purposes—in a public health service in the capital of *Rio Grande do Sul*, Brazil. After the medical examination, they were invited to participate in the research. All victims had already reported their partner to the police station and had the medical examination in the same place for legal reasons. The selection of women was by convenience, because only the women who were present on the day of collection were invited to participate. The number of participants was due to the abundance of data generated by qualitative research method. The women authorized their participation voluntarily in the research and signed an informed consent form. The identities of the participants were protected. All recordings of the interviews were numbered in a way that the researcher could no longer identify them later.

### 2.2 Characteristic aspects

The collection site was held in the psychosocial room, where the woman receives guidance on possible referrals. In this room, the interviewer and the participant were alone. It is a welcoming space where victims are heard and encouraged to reflect on: their situation, the reasons for the violence, the behavioral and emotional patterns that contributed to the occurrence of the aggression, and the social support network of each victim that could assist them at this time, among others. First of all, the victim safety is prioritized, removing her from places of risk, if necessary, away from the offender—Maria da Penha Law (Brazil, 2006). Also, counseling on possible legal procedures regarding assets, custody of children, among other information is provided.

### 2.3 Procedures for data collection and analysis

Data collection was performed by a psychologist researcher. The women answered a questionnaire on sociodemographic data. Later, they participated in a semi‐structured interview of OPD‐2 Clinical Interview that was recorded and transcribed. This interview is composed of five axes with themes that should be explored: assessment of domestic violence, interpersonal relations, conflict, structure, and mental and psychosomatic disorders (OPD Task Force, 2008).

**Figure 1:**
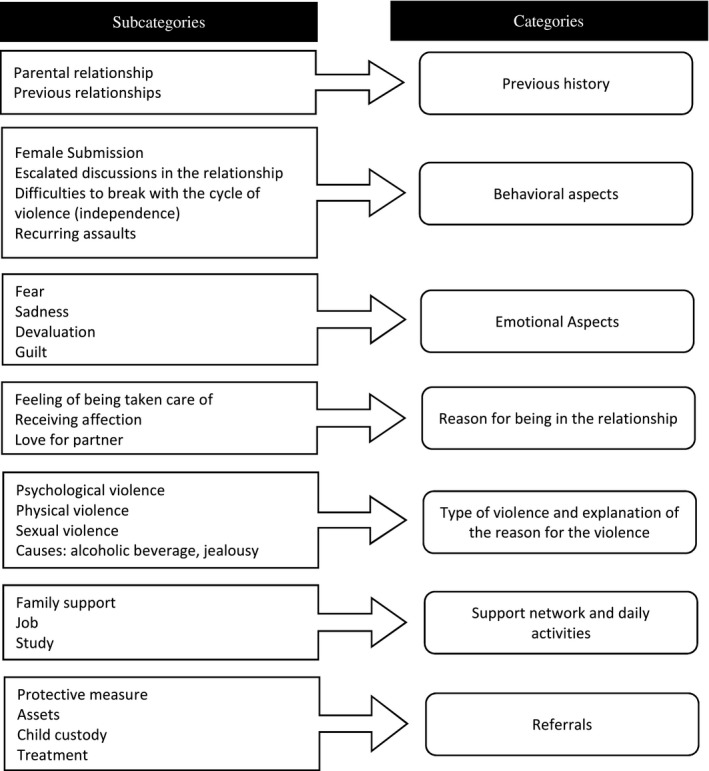
Categories of the study

A descriptive analysis was done to characterize the sample. The analysis of the interviews was carried out by two researchers who were independent psychologist. Then, the interviews were read incessantly. As proposed by Bardin (1994), the categories of analysis were created by a posteriori and the content analyses of the categories were performed. These categories were divided according to the thematic modalities of the subcategories identified in the interviews (Figure 1).

We subdivide the discussion in categories, contrasting the results with the existing literature pertinent to the theme. In addition, the classic scientific production of the domestic violence and psychodynamic functioning, international studies, and the investigations of local researchers on domestic violence, such as Lisieux E. de Borba Telles (*UFRGS*—Universidade Federal do Rio Grande do Sul) and Denise Falcke (*UNISINOS*—Universidade do Vale do Rio dos Sinos), were also used. These researchers are local references on the subject, since domestic violence has local social attributes to be considered.

## 3 HYPOTHESES

Hypotheses were created for each category. It is believed that cycle of violence is permeated by:

- Previous history: has transgenerational factors of violence and violent traumatic experiences in the victims;
- Emotional and behavioral aspects: feelings of dependence and fear of loss of the object because of their diffused identities, difficulties of mentalization, relationship instability, isolation;
- Type of violence and explanation of the reason for the violence: persistent psychology violence and alcohol abuse by partner; and
- Referring: protective measures and lack of treatment desire.

## 4 RESULTS

### 4.1 Characterization of participants: sociodemographic data

Ten woman victims of domestic violence participated. They were mostly white women (*n* = 7), with a predominant age of <35 years (*n* = 6), and half of them did not have a religion and had completed high school (*n* = 5), with income between 1 and 2 monthly minimum wages (*n* = 6). Among mates, the majority were white (*n* = 6), with primary education (*n* = 4) and income between 1 and 2 monthly minimum wages (*n* = 7), and half did not have a religion (*n* = 5). Most women did not use substances: alcohol (*n* = 9) or drugs (*n* = 8). However, half (*n* = 5) of the partners presented alcohol abuse, and most did not use drugs (*n* = 7; Table 1).

**Table 1:** Demographic data

Category	Subcategory	Women	Men
Age	18–20 years old	3	1
	26–30 years old	1	2
	31–35 years old	3	2
	36–40 years old	0	2
	41–45 years old	0	1
	46–50 years old	1	1
	51–55 years old	1	0
	56–60 years old	1	1
Race	White	7	6
	Black	2	3
	Brown	1	1
Scholarity	Incomplete elementary school	2	4
	Complete primary education	1	2
	Incomplete high school	1	1
	Complete high school	5	3
	Incomplete higher education	1	0
Income	None	2	1
	Less than 1 minimum wage	1	0
	Between 1 and 2 minimum wages	6	7
	Between 7 and 11 minimum wages	0	1
	More than 12 minimum wages	1	1
Religion	Atheist	5	5
	Catholic	3	2
	Spiritism	1	1
	Afro‐Brazilian	1	1
	Umbanda	0	1
Addiction	Alcohol	1	5
	Drug	1	0
	Tobacco	1	1
	Cocaine	0	1
	Tobacco and cocaine	0	1

Note

Income: 1 minimum wage is a basic remuneration for a worker. Religion: Atheist is the person who do not believe in God. Spiritism is the person who believe in life after death. Umbanda is the person who has several cults influenced by Indians.

The type of relationship was variable, four women had stable union, three were dating, two are separated from their partners, and one of them had a virtual relationship with only one personal encounter (Table 2). Regarding the care of the parents, half of them (*n* = 5) reported that they experienced, as children, fights and parents' discussion frequently, but they evaluated their parents as loving and caring.

**Table 2:** Relationship

Category	Subcategory	Participants (%)
Type of relationship	Date	1 (10%)
	Dating	3 (30%)
	Stable union	4 (40%)
	Divorced	1 (10%)
	Separated in less than 6 months	1 (10%)
Time of relationship	Less than 6 months	1 (10%)
	Between 6 months and 1 year	3 (30%)
	Between 3 and 5 years	1 (10%)
	Between 6 and 10 years	3 (30%)
	Between 16 and 20 years	1 (10%)
	Between 21 and 30 years	1 (10%)

### 4.2 Categories

The analysis of interviews and category findings is listed below. Some aspects are overlapping in more than one category, because there is no way to isolate the dynamics in independent thematic modalities.

#### 4.2.1 Previous history

In recalling the *parental marital relationship*, four participants (participants 3, 5, 6, and 7) comment on *good relationships*, as represented by participant 5: “Yes, yes, until today, they are married to 38 years old, never quarreled.” In contrast, participants 8 and 9 lived only with their mother, participant 8 states: “My mother was separated from my father… I raised was my mother. My father always wants other woman and forgets the children. I was very happy created by my mother, she fought to create me and had 4 brothers,” and participant 9 says: “I never had a father.” Participants 2 and 10, however, remember *conflict with their parents*, as observed in participant's speech 10: “She abandoned me because my father drank too.” And, some others do not bring experiences in relation to the conviviality between their parents (participants 1, 4, and 7). Finally, participant 6 was the one who sustained the house since the 13 years of age, because the parents were separated: The mother abandoned her when she left with a lover, and the father was an alcoholic and did not care about the care of the children.

About their life histories, it is verified that in their *previous relationships* they suffered aggressions (participants 1, 2, and 5). Participant 1 reveals about the previous partner: “It was terrible too, I separated in the Forum, it is the father of my daughters.” In the same way, participant 2 reveals: “The other partner I had, thank God already died, he beat me” and participant 5 reveals: “No one likes, no one, no one, not his family, nobody, because he is a aggressive person, only he thought he was right, my ex‐partner.” On the contrary, participants 9 and 10 tell about positive relationships, an example is participant 10: “We live together, he is very good, he does not lack anything.”

#### 4.2.2 Behavioral aspects

About that, it is reflected on the behavioral aspects of partner during relationship. It is perceived that prevalent characteristic in the participating women is *submission* to dominant “power” of the man, since they remain in the relationship and support the violence and frustrations, also coming from the parental model: submission versus independence.

It is not possible to isolate this thematic field from the previous one, as in the following example: Participant 3 claims: “My mother was always very independent, she always worked out, she always, always, understood? And she always gave me this, which never depends on man, I have to always have my independence (...), then that independence that I have always had all my life, suddenly I have no more, I become dependent, both is that he left at dawn on Wednesday, I screamed so much, so much, that he was scared.” It may be noted the need to have time of your life, such as financial independence gained by the participants 2 and 6. On the other hand, there are those submissive to fellow wills (participants 1, 3, 4, 5, 7, 8, 9, and 10), as commented by participant 2: “I don't know (...) my mother was one of those who asked my father that way: father gives me 10 reais to buy 1 kilo of rice? then I buy the kilo of rice, I had to give back the rest of the money to dad, I found it, I think it's awful.”

In previous history, it was evident that the *beginning* of their *conjugal relations* took place in a harmonious way, being able to carry out joint activities, as observed in participant 2: “No, in the beginning, you are looking at the picture when we were well” and participant 5: “We went out, had fun, we went to the bar, we talked, we went.” It is observed that with the passage of time, socializing couples began to have *discussions* and demonstrations of *jealousy* as participant 3. It reveals that jealousy was so intense that his partner did not allow her to work: “I work at home since I married him, I had to stop working because he did not accept.”

One question raised by three women is that such *difficulties intensified* after they had moved in with their partners (participants 1, 7, and 10). The reasons for changes have given up by problems that have related to marriage union or labor issues as the participant 1: “I moved to Gravataí, because he had a problem, what happened to him at that time my house here in Porto Alegre, near the police station. I sold there, now I moved to Gravataí, I've been in Gravataí for 18 months (...) he got me at the police station in Porto Alegre (...) I threatened and I was horrible on this side.”

In face of behaviors experienced in the relationship, participants 2, 3, and 5 reported difficulties in achieving an attitude to *break the cycle of violence*: Participant 2: “I don't do anything, I will do what?.” Participant 3: “Then I stopped living, because then I, my mother lives on the third floor, I in the second, I couldn't go up to my mother anymore. But what are you going to do there? So he thought, I'd go to my mother to talk to someone on my cell phone.” Participant 6: “I don't know where else I, you know? where that I, where, where, I had to have the cut, my head is totally confused now.” When there are attempts to independence, the partner rebels, as experienced by the participant 6, who despite making some moves, no success: “Twice I've left home, since rented an apartment, it was back and tuck in from the apartment.”

Some women reported that aggressions were *recurrent*, both physical and verbal (participants 1, 2, 5, 6, and 10). Still others have pointed out that they were also physical aggressors as a form of *defense* (participants 1, 2, 4, and 6), or they ran away in the face of partner's aggression—fleeing (participant 5). Participant 8 states: “What he did to me? because my mother didn't raise me for him to beat me up, and he picks up from anyone, I would not accept that my mother beat me, now I'll accept from shit hit me,” using the law to protect themselves and put a limit.

#### 4.2.3 Emotional aspects

Participants 1, 3, and 9 indicate feelings of *fear* in relation to what was experienced and feelings of incapacity in the face of the situation. Participant 1 says: “For fear, he is very ‘barreiro’, very bad, very bad, only that my life has become a hell, he threatens me every day, as if it were terrorism.” In the same way, participant 5 said: “I slept with an enemy like this, I was afraid of death.” There were still aspects of *sadness* in participants 4, 5, and 10. Participant 4 said: “I was very nervous, I cried, I was worried.” Participant 10 said: “I cry every day.” In addition, participants 4 and 5 respectively bring feelings of *devaluation* in front of themselves: “He manipulated me I felt like a poor person, I felt the worst, I felt ah I did not even, I felt worse, nobody can help me,” “I felt like crap, it made me feel like crap.” It is also possible to raise in participant 5 feelings of *guilt* in relation to the fights and attitudes of the comrades: “I feel guilty (...) it is something that hurts me to do this for him, I feel sorry for him,” referring to guilt in complaint.

In addition, there are interviewees who feel *guilty* (participants 4, 6, 8, and 9), as shown in the speech: participant 4 said: “So, everything that happened was my fault, I was with the boy, if I had not stay with he, involved me with him, impregnate (…) I was feeling guilty, very, very, very much, if I knew that all this would happen, I would have turned into a hurricane, I had not even looked at it, passed straight.” Participant 6: “It's so oh, I'm sorry today for not the first time, so oh, that he had some crisis, so oh, that he had said did not arrive, give limit, I repent a lot yes, so oh, I should have said no, you there, I'm here and you get it, I regret it a lot, if I did not do it at the beginning, I left it, I did not stop at the beginning.” Participant 8: “I feel guilty for believing all this.”

#### 4.2.4 Reason for being in the relationship

A common feature of the study participants is the permanence in the relationship, even qualified as abusive and violent. They remain due to the feeling of *being cared for and receive affection* (participants 1, 3, 4, 5, 7, 8, and 9) and/or because they *felt love from their partner*, stressing these violent moments (participants 2, 5, and 8) like the participant 2: “Because I liked him, was afraid of losing him” and participant 5: “That thing so I will not have anyone to help me.” Some had the hope that the partner could change his attitudes (participants 3 and 10), participant 3: “Hope he changed, because after all he did, he would fight all night, but we got it right.”

Another issue that can be raised that influenced the continuity of the relationship refers to health issues (participants 1 and 5), both of women and their children, observed in following statements: participant 1: “I have problems in the knees, a disease that has no healing in the bones, hence I am leaning against it. I could not drive the van. He did the races for me.” Participant 5: “I was already depressed, because of this, that I was not even working.” Participant 6: “Her well‐being (daughter) so oh, it's been 6 months now that she's doing this treatment right.”

#### 4.2.5 Type of violence and explanation of the reason for the violence

The majority reported having suffered *physical* aggression from partners (participants 1, 2, 4, 5, 6, 7, 8, and 10), *psychological* violence (participants 3, 4, 5, 6, and 7)—humiliations and insults—with case threat (participants 1 and 9), or in case of separation (participant 6). Participant 6 exemplifies this question: “I will take our daughter if you separate from me, you will never see her again, I will not appear again, I will not see her again (cry)” or participant 3: “such a knife to cut meat, stab you all, then I'll kill you, I'll show you, if you do not stay with me, you will not stay with anyone in this life.” One of them reported *sexual* violence of a seductive boy from a virtual relationship (participant 9). Participant 9: “I didn't know that, he used drugs, he was aggressive (...) He put a gun on the side of the bed, I've never seen a gun in my life.”

Regarding the *verbal threats*, participants 1, 2, 3, 4, 5, 8, 9, and 10 suffered constantly, as exemplified in the following lines: participant 1: “Because you are old, I'm sick of this old, because I can't stand the voice of this old woman, he was already mistreating me understood, little by little.” Participant 5 “After about 2 months, when we were together, he showed me who he was (...) we fought, he used to scold me for a lot of things, he used to spit on me, he used to call me junk.”

For participants 1, 4, 5, and 9, the aggressions became physical as revealed: participant 1: “Bah, it was horrible, it was very ugly there (...) I was horrible on this side, this time I was disfigured (...) he drops me and had the courage to sit on top and I get on the same side all the time, I leave horrible.” Participant 2: “it's been almost 2 years, I'm trying to get my partner out of the house, because he drinks too much, and now he starts assaulting me, he bounces on me with a knife.” Participant 5: “He knocked me, gave two punches and I managed to get out running like that, between the room and I knocked the child on the ground, that I purposely threw my daughter on the ground, to him at the moment he can get up right. He brushed my hair so he could get it, and it was the moment I got out of the door, and I ran away.”

The *causes* pointed out by the women participants were practically unanimous: use of *alcohol* by the partner (participants 1, 2, 9, and 10) and/or *jealousy* of the partner (participants 2, 3, 4, 5, 6, 7, and 8). She could not talk to other people, especially men, or wear some specific clothing. Example of participant's talk 10: “Yes, yes, with a drink, he spent a lot of money, every weekend.” Participants 8 and 9, however, reveal the use of marijuana and cocaine by their partners, as reported by participant 8: “He smelled cocaine and marijuana.”

#### 4.2.6 Support network and daily activities

The support network and work and study activities are variable in these women. Some women reported having *family support* in crisis situations (participants 3 and 4) and help from *coworkers* (participant 7). However, some do not feel they have anyone other than their partners (participants 1, 5, and 10), as in participant 5: “He has an abusive relationship, he gives me psychological pressure, nobody will help me, that no one likes me, you know, that thing like that, that I will not have anyone to help me, right?” Or they have only *children* as the only source of fulfillment in their lives (participants 2, 6, and 8). The response of participants 7 and 9 is highlighted when asked about who helps or cares for her when necessary: “Myself,” “I and God,” respectively. Three of them said they did not want to disturb or worry their relatives (participants 5, 7, and 8), so they did not seek help, isolating themselves from the others. Some women are without paid *work*, not always meaning financial dependency, but without a work activity in which they feel useful (participants 1, 8, and 9). Also, the *study* is an activity practiced by only one participant (participant 6) and desired by some (participants 7 and 8).

Another factor to be considered relates to the feeling of lack of acceptance (participants 1 and 4) by the responsible authorities or sectors in being able to report the aggressions they suffered: participant 1: “When I went there at the police station (...) he (partner) arrived at the same time and the guy (police officer) is sorry. I don't know what it was, he said: why you no talk with other? Sit there. And it doesn't give anything.” Subsequently, when making a phone call denouncing his partner because he already had a protective measure and was at home, participant 1 also says: “He was very stupid (police). He gets really angry, I have so much to do, and I have to come here.” Participant 4 also points out this difficulty when registering a complaint about the verbal/ psychological violence she suffered: “And the policeman said, I do not even want to hear the recording! I did not understand until today.”

#### 4.2.7 Clinical and legal referral

Most of the women (*n* = 8) made the referral of the *protective measure* guaranteed by the Maria da Penha Law (Brazil, 2006) and remained in their homes, while the partners sought another place to live. However, with regard to *assets* in common with the partners, one of them requested a measure and seizure of the care that the partner stayed (participant 1). In relation to the *children,* one of the women intends to prevent contact with the father (participant 3), there is one interviewee who awaits the judicial decision to regulate visits for the children to have contact with the father again (participant 4) and, unlike, there is another who cannot see the children who stayed in the partner's house (participant 10). About motivation for psychological or psychiatric *treatment*, few have shown interest; only three would like to return to treatment, since they have had prior treatment experience (participants 5, 6, and 9).

## 5 DISCUSSION

**Figure 2:**
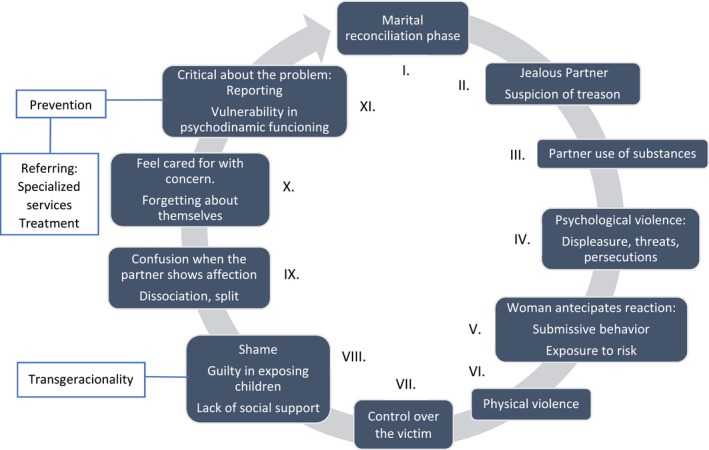
Stages of cycle of violence

We believe that there is a cycle in the context of domestic violence, from a traumatic perspective, which will be explored (Figure 2), confirming the hypotheses.

This figure shows an outline of the cycle of violence algorithm proposal. At stage number I, there is what is called the honeymoon phase of the relationship, when the couple is still in harmony. However, according to the perception of the female victims, the partners are very jealous, since they suspect betrayal (Stage II), in order for the partner to calm down. A partner who turns out to be violent usually abuse substances, mainly alcohol (Stage III), according to the victims' perception. As a consequence, lack of control of the partners' violent impulses and emotions that he already possesses in a latent form that will then pass to actions. Threats, persecutions, and psychological violence begin to happen (Stage IV). With that, the women start anticipating violent reactions, and end up behaving in submissive ways to please their partners. Thus, they remain in the relationship and are continually exposed to risk (Stage V).

At the Stage VI, physical aggression will take place, as partners form of reestablishing control of the relationship (Stage VII) with progression of the cycle of violence and greater impulsivity from the men. Dealing with this situation, at the Stage VIII the victim feels ashamed to expose themselves and ask for help to break the cycle of violence. Consequently, there is the isolation due to the woman's shame and jealousy of the husband that prevents her from working or meeting with friends and family. Also, they feel guilty for exposing their children to situations of violence, in which can have transgenerational effects by promoting a parental model that is being repeated by them and will be repeated by their children. However, in different stages of the cycle of violence, some women manage to find the strength to seek help.

According to the report of the National Council of Justice, the number of protective measures during the year 2017 increased considerably. Throughout Brazil, 236,641 measures were issued in 2017, an increase of 21% compared to 2016. Specifically, Rio Grande do Sul was the State that issued the most protective measures based on the Maria da Penha Law in 2017 (CNJ, 2018). Despite this, in 10 years, there is a 6.4% increase in Brazilian femicides (IPEA, 2018).

However, when woman thinks about taking action to protect themselves and the partner shows affection again the victims become confused by the care they are receiving. Dissociation and division occurs when they dominate the minds of victims all aspects of violence are disregarded or even seem to disappear (Stage IX). Besides that, the partner's concern expressed as jealousy and control over the victim are misunderstood by women who feel cared for. The victims forget about themselves and dedicate most of their time taking care of others as a way to feel cared for (Stage X). When the victims become aware of the violence to which they are subjected to, obtaining insight of the cycle of violence as a whole, they find courage to report the partner. It is in this moment of rationality that occurs the possibility to break the cycle of violence. When victims recognized all their suffering, they finally seek some kind of support and assistance breaking the pattern of isolation and shame. In any case, such victims present a vulnerability in the psychodynamic functioning that needs to be strengthened to break the cycle of violence (Stage XI). However, not all victims continue with the legal procedure because they reconcile with the aggressive partner and start to relive the cycle of violence sometimes without realizing that they are returning to Stage I.

### 5.1 Sociodemographic data

The study was conducted with women victims of domestic violence who sought out public service for medical examination. Most were white, aged 18–35, completed high school education, and with income between 1 and 2 minimum wages. Their partners were also whites with predominance of elementary level of schooling and income also between 1 and 2 minimum wages. This profile is observed in different studies, as risk factors associated with domestic violence (Audi, Segall, Santiago, Andrade, & Pèrez, 2008; Silva, Falbo, Figueiroa, & Cabral, 2010).

However, these data show consistency with the local study of 751 couples that mapped marital violence in the South of Brazil (Rio Grande do Sul state). The authors pointed out that higher schooling was associated with a lower level of violence, since it aids in talking and negotiation for conflict resolution; the sample of this study presented median schooling and incomplete high school education. Income was not a significant factor, since there were high levels of violence in both contexts, despite the majority being of low income. Lower ages were associated with higher levels of violence (Falcke et al., 2017).

### 5.2 Previous history

The present study showed that participants had differences in their previous history, some of them lived traumatic experiences while others healthy experiences. In the literature, adverse experiences in the family of origin are not a determinant factor for the pattern to reoccur in adult life (Yoshihma & Horrocks, 2010). Personality disorder in women is considered to be a mediating variable in relationships between experiences in the family of origin and conjugal violence (Ehrensaft, Cohen, & Johnson, 2006; Madalena, Carvalho, & Falcke, 2018). The participants who grow up in a violent environment could repeat parental patterns and developed a more serious pathology, but issues in the family origin are not the only determinant in current cycles of domestic violence.

In stress and crisis situations, participants presented difficulties to make significant changes in their daily life to break the established cycle. In this sense, it can be understood that the aggressions suffered, recurrent or isolated, are characterized as stressful traumatic situations in their lives, the trauma overwhelms the capacity of the ego to process anxiety and pain (Zimerman, 2001) producing disruption or distortion (Benyakar, 2002). Thus, the use of defensive mechanisms, such as dissociation, repression against traumatic memories, is common in these cases as was observed in the participating victims.

Attitudes toward trauma/violence demonstrate a lower or less structured personality associated with unsafe primary psychic representations. It is observed in the stories of the victims that experienced fights and discussions during their development, and these characteristics can be a transgenerational aspect also having influence when choosing partners (Coimbra & Levy, 2015). In these cases, there is identification with the victim's role and a repetition of parental patterns. Several studies point out that involvement in contexts of family violence as a victim or being a direct witness increases the possibility of establishing a violent conjugal relationship in their own adult life (Falcke, 2006; Milner et al., 2010; Zacan, Wassermann, & Lima, 2013). Another study found that women who experienced domestic violence, whose mother had been beaten by their father, were three times more likely to suffer domestic violence than women who did not witness such aggression (Adjah & Agbemafle, 2016).

### 5.3 Behavioral and emotional: cycle of violence maintenance

Anthropologically, when thinking about the construction of the phenomenon of violence against women, we must highlight the historical and cultural factors involved, in which domestic violence against women is trivialized and naturalized in today's society and among different cultures. In this sense, men's desires and wishes must dictate over submissive and subordinate women, generally having inequalities in different contexts such as social, work, family. Domestic violence against women studied here has issues of great magnitude, since the aggressions were recurrent and the women remained silent, perhaps not only for psychological reasons (Bins et al., 2015).

The aggressions often start from a repetitive and recurring pattern of control and domination. This control and the control over the life of the victim is called *Stalking*; defined as the perpetrator's imposition of unwanted approach and communication behaviors that induce fear in the victim. It involves intrusive, obsessive, and unwanted acts. These characteristic are common behaviors related to psychiatric disorders (Bins et al., 2015). These aspects were very present in the stories of the interviewed women, in which the partners controlled their lives, preventing them from living.

Some of the victim's reactions to violence were as follows: defending themselves of the aggression, fleeing, isolating themselves, and/or grieving. However, most of the time they felt intimidated to break the cycle of violence. The victims remained in the relationship due to fear of future aggression or of risking their lives or their children's if reporting (Zacan et al., 2013). However, there is a feeling of guilt for exposing their children to situations of violence, due to the considerable effects on mental health and performance in school activities (Carneiro et al., 2017).

Also, victims often do not break the cycle or report the partners due to difficulty in recognizing their interactions as violent (Garcia et al., 2008), especially in cases of psychological aggression. Emotional abuse, studied by Bins (2012), is considered one of the most difficult types to be identified, but increasingly studied and associated with the development of psychopathologies.

In addition, it can be hypothesized that trauma, due to constant violence, causes changes in structural functioning and intrapsychic conflict (OPD Task Force, 2008). Violent actions damage the ability to think and comprehend (Souza, Martins, & Araújo, 2011). According to the theoretical and clinical reference of the theory of mentalization of Bateman and Fonagy (2010, 2016), it is observed that the participants have difficulties in mentalization. In this case, hypermentalization occurs due to trauma, in which excessive interpretations of the mental states of others occur, and the subject distorts and interprets reality in a wrong way, a vulnerable mentalization. The predominance of unreflective, rigid, and automatic assumptions sustained with the unjustified certainties of internal and other mental states is common. Moreover, they focus on their external aspects too much (Sharp et al., 2013, 2011, 2016). In this way, there is instability in the relationships with psychological conflict focused on emotional dependence on the other.

As a consequence, they become submissive, and abandon their desires and projects due to their partner's life and there is difficulty in having a sense of identity. Thus, they present a diffused identity with difficulties to describe themselves over time in a consistent and coherent way, as observed in the participants. According to Kernberg, Selzer, Koenigsberg, Carr, and Appelbaum (1991), identity diffusion is defined as the lack of integration of the concept of self, from the patient's subjective experience of chronic emptiness and self‐perceptions and contradictory behaviors.

Concerning submission to psychological violence by the participants, most of the participants in the study mentioned that such violence increased their feelings of incapacity. This fact is pointed out by Guimarães and Pedroza (2015) as an abandonment of a self‐sense of dignity, since this dignity is denied by the other, the aggressor. According to the Maria da Penha Law (Brazil, 2006), psychological violence is any action that will cause emotional damage and decrease self‐esteem, that harms and disrupts development, or that seeks to degrade or control the actions of women and their behaviors, through threat, embarrassment, and humiliation.

Regarding the emotional aspects identified, the predominant feelings pointed out by the participants were fear, anxiety, anguish, and even guilt, affirming the complex unconscious dynamics and the contradictory feelings present in the relationships. In relation to the defense mechanisms, it is observed that the use of dissociation prevailed as a way of retaining an illusory control in the face of helplessness and lack of control; the split, since they perceived the partners as good objects or bad and aggressive; and denial, in which they avoided awareness of the difficult aspects of relationship and past history. Some showed somatization, which is characterized by the conversion of affective states into physical symptoms (Gabbard, 2016).

### 5.4 Type of violence and explanation of the reason for the violence

According to Bins Telles and Panichi (2015), violence against women is a multicausal phenomenon: (a) community factors: poverty, unemployment, family isolation; (b) factors of society: naturalization of violence to resolve conflicts, male domination, stereotyped gender roles; and (c) factors of the aggressor: use of substance, transgenerationality of violence. These issues were observed in the participants' stories.

In regard to the causes of the aggressions, it is consistent with reviewed literature, such as the use of alcohol and drugs, jealousy of the partner with suspicion of treason, as reported by the participants (Zacan et al., 2013). The use of substances by their current partners made the possibility of dialogue and understanding even more difficult factors that concluded in different forms of violence: threats to physical integrity, physical aggression, psychological, and even sexual violence. However, another discussion raised by Falcke, Boeckel, and Wagner (2017) states that the phenomenon of violence is interactional, with high rates of mutual and equal violence between the partners. In the literature, it is pointed out that the women practice more psychological violence, while men practice more physical aggression.

Still, Chauí (2003) complements affirming that the aggressor denies a sense of purpose, wishes, freedom, and responsibility of the victim, treating women as objects, paralyzing them so much the victim feels like “trash.” According to Saffioti (1999), violent actions will occur when the aggressor perceives that he is losing power or feels incompetent in front of the other, attacking and seeking to destroy capacity of choosing, a hypothesis experienced by a participant. In some interviews, in which there are attempts of independence, in these moments partners present aggression when feeling threatened of losing the object (woman). Thus, in the face of such traumatic events experienced continuously, relationships were maintained even if they suffered or felt angry.

### 5.5 Support network and daily activities

As justifications by the participants for staying in the relationships, we can point out the psychosocial difficulties, such as lack of social support and the community's protective network, financial dependence for children's needs and household maintenance and food expenses (Amaral, Vasconcelos, Sá, Silva, & Macena, 2016; Bins et al., 2015), and lack of information and counseling on legal procedures, to the public health networks, to shelters (Cunha & Pinto, 2015).

On the other hand, other participants report having financial independence, which suggests that there are other elements in staying in the abusive relationship, such as low self‐esteem, allowing themselves to be controlled and even manipulated by their partners, feeling “imprisoned” in the relationship, adapting to that reality. In addition, it is noted that some have remained in the relationship because they feel at least their receiving care from someone. In this sense, it reinforces the hypothesis of the establishment of an insecure attachment in previous history, with ties a dependence in relation to the other, common in women who suffer violence (Montero, 2001). Another hypothesis is that this “care” may be due to the sadomasochistic aspects of the aggressor, in which this characteristic is considered to be a high risk for aggression, even fatal (Bins et al., 2015).

In this way, they lose interest in social interactions and other daily activities (Carneiro et al., 2017; Falcke & Féres‐Carneiro, 2011), diminishing affection, in relation to both negative and positive emotions, which makes it even more difficult to social contact and future planning (Barlow, Allen, & Choate, 2016). Only after being able perceive and work on these dynamics do they begin to see other perspectives and start activities such as studying and working, as observed in participants or viewing such activities in another way, toward independence, freedom, and autonomy.

One study presented evidence that higher frequency of social contact was associated with less injury to the victims (Kane et al., 2016). However, the participants in this study reported not seeking family and friends so as not to disturb them, isolating themselves more and more, as well as not working or doing other activities.

Access to the support network and specialized services is indispensable to assist such women in reflecting on their lives, their choices, and ways of breaking the cycle of violence. Participants reported that when they went for help they went to family or friends at work. The lack of social support and in some cases the difficulty in asking for help, as well as a prejudiced attitude from those who should show support, resulted in increased suffering and the progression of more serious aggressions (Meneghel et al., 2011), as observed in the study, in which there is a progression of psychological violence to physical (Bins et al., 2015). Some of the women pointed out that they do not want to bother anyone, so they remain quiet and handle the situation on their own.

### 5.6 Referring and prevention

It is noteworthy that all participants in the study had already filed a report of aggression at the police station, but the majority did not have information of security and protection rights that they are entitled to according to the Maria da Penha Law (Brazil, 2006). Access to information is still a flaw in the community, and this is an important point for the prevention and eradication of domestic violence (Cunha & Pinto, 2015).

According to Signori and Madureira (2007), the situation of violence in a relationship is also aggravated by the shame women have in reporting it, the lack of educational means, and lack access to legal information and of assistance and protection. In the participants, the aggressions were recurrent, only reporting it when it reached their limit of frustration. In this sense, the participants demonstrated confidence in the protective measures as a way to ensure their protection, an initiative to break the cycle of violence. However, few of them had the desire for treatment, to change these dynamics, maybe because of the difficulty of mentalization in reflecting and perceiving themselves or the other in the relationship, the future of their choices, and the consequences of their actions.

In addition, another issue raised by Signorelli, Taft, and Pereira (2018) refers to the existing gap in the implementation of Brazilian public policies, despite the benefits of including women in these services and listening to them. The psychosocial reception of the service has helped the victims to reflect on the choices and attitudes in their lives; however, there are still many deficiencies in the public service that need to be discussed: Not all women are listened to and given guidance due to the dynamics of the service, since only the medical examination is mandatory for the report, so the focus of process remains with physician, the psychosocial team can only approach the victim after the examination. Thus, we suggest a reformulation of the dynamics of the services for the better care of these women, taking into account the needs that go beyond legal and police guidelines, because only then will it be possible to promote a significant change in the cycle of violence. We suggested the implementation of waiting room groups, for a more inclusive support network, among others.

With this, we can think of prevention from three perspectives: primary, secondary, and tertiary. Primary prevention is more focused in a macro level, when violence has not yet occurred. It is important to note that in the case of anthropological issues of power and gender, women have a more submissive and adaptive attitude toward abusive and coercive behavior (Cortez et al., 2010). In this sense, it is possible to facilitate access to the information and procedures regarding domestic violence supported by media and communication networks on protection rights; to eradicate the sociocultural pattern of stereotyped gender roles (Brazil, 2006; Cunha & Pinto, 2015).

Secondary prevention is when it is happening, so what can be done? From this perspective, it is thought at the micro level, with the strengthening of the individual resources of the victims to assist in the management of the traumatic event. This proposal is based on therapeutic listening and specialized health services that promote psychological changes in these women (Garland, 2015). However, this is not a right entitled in the assistance of women insured under the Maria da Penha Law (Brazil, 2006). There are legal guidelines that ensure mechanisms such as (a) social, with the registration on public programs with housing benefits, maintenance of employment bond; (b) health, with free contraceptive methods for cases of sexual violence; (c) public security, guaranteeing victim protection; and (d) tertiary prevention, regarding aggressions that occurred in the past. This perspective is similar to the secondary interventions, which we seek to emotionally strengthen the victims. Also, it is suggested the creation of a program for aggressors, an example to those done internationally (Barin, 2016); after all, these aggressors are also suffering and have their difficulties (Bins et al., 2015).

## 6 LIMITATIONS

The biases were controlled as much as possible. This research is a qualitative research, in which the researcher's subjectivity can measure results; thus, all interviews were analyzed by two independent judges. And we base the data on the guidelines of Consolidated Criteria for Reporting Qualitative Research (COREQ; Tong et al., 2007) so that there would be greater methodological consistency.

In addition, the interviews were performed only from the audio transcription, so non‐verbal behavior of the patient was not evaluated. Sometimes, the expressiveness was kept in report, such as crying, anger, among others. Also, part of the interview was dedicated on counseling the victims on legal procedures.

Regarding the collection place, it was not a clinical setting, but a police station with the intention of filing a report against someone and also seeking protection. Thus, in some cases, there was a high amount of defense mechanisms or even dissociative defenses that could distort the facts. Regarding the sample number, there are only 10 women, but the data saturation criterion was used, so it is evident that the data collected are consistent with reality.

## 7 CONCLUSIONS

The present study collaborated with the understanding of the psychodynamic issues of the cycle of domestic violence against women. The dynamics of violence reported by the interviewees is observed in other studies on this subject. However, there are few studies that focus on aspects of psychodynamic theory on the cycle of violence. It is necessary to understand this cycle of violence to create more effective coping mechanisms. What is innovating in this article is the construction of this cycle of violence. Adverse experiences were observed during the development of the victims, generating feelings of dependence and fear of loss of the object because of their diffuse identities. Even when the participants demonstrated a more integrated psychic structure, the trauma caused difficulties of mentalization, emotional dependence, and relationship instability. Such understandings have enabled us to reflect on the definitions and patterns of violence against women emphasized in the literature, identifying the relevance of this clear conceptualization to (re)affirm the breadth and diversity by which such violence can express itself.

Tackling violence involves new ways of offering support to women, allowing a space for listening and working not just for the legal procedures, but as an attempt to stop the cycle of violence. Therefore, a specific assessment of the patients seeks to identify the psychological and social resources and obstacles of the patient, her personal understanding of the symptoms, and the characteristics of the psychodynamic functioning. Likewise, it is important to extend the educational work seeking the deconstruction of cultural and social standards instituted in relation to the gender that authorize and naturalize male domination.

Many issues still need further studies, specifically in psychodynamics perspective about the cycle of violence. However, the research seems to collaborate with greater evidences on the subject, especially in the context of the metropolitan region of the South of Brazil (Rio Grande do Sul, state). It is pointed out as limitation the fact that the participants came from police station, whose victims filed a report to the police, different from those who are still “imprisoned” inside the family. In addition, the present study has a cross‐sectional investigation design so lacking the long‐term feedback from the participants. Future studies investigating a longitudinal sample would have a greater potential to understand the complexity of the phenomena involved in the cycle of violence against women increasing understanding in this very important field.

## CONFLICT OF INTEREST

There are no conflicts of interest.

## ETHICAL APPROVAL

The study received the approval of an institutional ethics committee and authorization for data collection.

## INFORMED CONSENT

The victims were invited and authorized their participation in the research by signing the informed consent form.

## OMISSION

All data that could identify participants were omitted.
